# Quantitative Trait Loci Analysis Based on High-Density Mapping of Single-Nucleotide Polymorphisms by Genotyping-by-Sequencing Against Pine Wilt Disease in Japanese Black Pine (*Pinus thunbergii*)

**DOI:** 10.3389/fpls.2022.850660

**Published:** 2022-04-05

**Authors:** Tomonori Hirao, Koji Matsunaga, Kenta Shirasawa

**Affiliations:** ^1^Forest Bio-Research Center, Forestry and Forest Products Research Institute, Hitachi, Japan; ^2^Kyushu Regional Breeding Office, Forest Tree Breeding Center, Forestry and Forest Products Research Institute, Koshi, Japan; ^3^Department of Frontier Research and Development, Kazusa DNA Research Institute, Kisarazu, Japan

**Keywords:** genotyping-by-sequencing, genetic linkage map, pine wood nematode, pine wilt disease, PWD resistance

## Abstract

Identifying genes/loci for resistance to pine wilt disease (PWD) caused by the pine wood nematode (PWN) is beneficial for improving resistance breeding in *Pinus thunbergii*, but to date, genetic information using molecular markers has been limited. Here, we constructed a high-density linkage map using genotyping-by-sequencing (GBS) and conducted quantitative trait loci (QTL) analysis for PWD resistance for the self-pollinated progeny of “Namikata 73,” which is the most resistant variety among resistant varieties of *P. thunbergii*, following inoculation tests with PWN. An S_1_ mapping population consisting of the 116 progenies derived from self-pollination of the resistant variety, “Namikata 73” (resistance rank 5 to PWN), was inoculated with PWN isolate Ka-4 and evaluated for disease symptoms. To construct a high-density linkage map, we used single-nucleotide polymorphisms (SNPs) identified by GBS based on next-generation sequencing technology and some anchor DNA markers, expressed sequence tag (EST)-derived SNP markers and EST-derived simple sequence repeat (SSR) markers, and genomic SSR markers. The linkage map had 13 linkage groups (LGs) consisting of 2,365 markers including 2,243 GBS-SNP markers over a total map distance of 1968.4 centimorgans (cM). Results from QTL analysis using phenotype data and the linkage map indicated that PWD resistance is controlled by a single locus located on LG-3, as identified in a previous study. This locus showed overdominant genetic action in the present study. With the confirmation of *PWD1* in two different mapping populations (present study and a previous study), the locus associated with this region is thought to be a good target for marker-assisted selection in *P. thunbergii* breeding programs in order to obtain high levels of resistance to PWD caused by PWN.

## Introduction

The conifer species *Pinus thunbergii* Parl (Japanese black pine) was widely planted in coastal areas of Japan to prevent land erosion and to provide protection from wind-blown sand and tidal waves. However, since around 1970, the spread of pine wilt disease (PWD) caused by the pine wood nematode (PWN) *Bursaphelenchus xylophilus* has become a chronic problem in pine forests in Japan (Kiyohara and Tokushige, 1971). Control measures for PWD have taken the form of breeding programs to select and develop more resistant pine varieties, and genetic analysis has been implemented to improve cost-effectiveness.

The first breeding project to develop pine varieties resistant to PWD was started in 1978 in southwest Japan (Fujimoto et al., 1989), and as the damage has spread, related projects have been promoted throughout Japan with the exception of on Hokkaido Island (Kurinobu, 2008; Forestry Agency Ministry of Agriculture, Forestry and Fisheries of Japan, 2010). In the first breeding project conducted from 1978 to 1984 in southwest Japan, 16 resistant clones were selected from 15,000 candidate *P. thunbergii* trees and were further developed as resistant varieties (Fujimoto et al., 1989). As of March, 2019, 171 resistant individuals have been selected from pine forests with severe PWD damage from all over Japan (Forest Tree Breeding Center, Forestry and Forest Products Research Institute, 2019).

*P. thunbergii* has 12 basic chromosomes (2n = 24) and an estimated genome size of approximately 25 Gbps (Neale and Wheeler, 2019). The reference genome of this species has not yet been determined. To date, several genetic linkage maps have been constructed using DNA markers (Kondo et al., 2000; Hayashi et al., 2004; Hirao et al., 2019), with the genetic linkage map identifying the locus for resistance to PWN in *P. thunbergii* by Hirao et al. (2019) being the only genetic study to date to identify resistance to PWD using genetic markers. The genetic linkage map was constructed using the genomic simple sequence repeat (SSR) and expressed sequence tag (EST)-derived single-nucleotide polymorphism (SNP) markers in resistant F_1_ families, and the locus related to PWD resistance, *PWD1*, was mapped on linkage group (LG) 3. It is necessary to accumulate more knowledge of the genetics of *P. thunbergii* in order to elucidate the mechanism of PWD resistance and to improve breeding success for resistance traits.

Through gene-assisted selection (GAS) and marker-assisted selection (MAS) tools, improved genetics and more cost-effective breeding programs can be developed. Furthermore, more effective genome-wide and high-density linkage mapping with more effective genotyping methods are needed to more accurately detect PWD-resistant loci.

The use of SNPs is advantageous over many other markers due to their abundance in the genome, their ubiquitous distribution throughout the genome, and their typical biallelic and co-dominant characteristics. Recently, new approaches, such as restriction site-related DNA sequencing (RADseq) and genotyping-by-sequencing (GBS), have led to the discovery of thousands of SNPs and the cost-effective genotyping of some crops (Davey et al., 2011; Andrews et al., 2016). Among the different approaches, GBS is the simplest and most cost-effective approach due to simple procedures to prepare the library and a high level of multiplexing capacity, as well as its effectiveness in organisms for which complete genome sequencing is lacking (Elshire et al., 2011). The GBS approach is widely used in many crops in breeding and molecular genetics studies, genetic diversity studies, trait mapping, and genome-wide association studies (Poland and Rife, 2012; He et al., 2014; Chung et al., 2017), and it has also been attempted in coniferous species with large and complex genomes (Pan et al., 2015; Hall et al., 2020). Identifying the genetic factors for PWD resistance is critical for the development of PWD-resistant clones of *P. thunbergii*.

In a previous study, QTL analysis for resistance to PWD in *P. thunbergii* was conducted using the F_1_ population of crosses between resistant varieties with a pseudo-testcross strategy (Hirao et al., 2019). Employing a pseudo-testcross strategy (Grattapaglia and Sederoff, 1994) for linkage mapping in a controlled cross between two outbred parents is widely utilized for mapping quantitative trait loci (QTL) in outcrossing species. However, the F_1_ population often displays many different types of segregation because outcrossing species are highly heterozygous. Some loci may have four different alleles between the crossing parents, generating four genotype classes in the progeny. Many others may also follow the F_2_ pattern in a 1:2:1 ratio and the backcross pattern in a 1:1 ratio (Lu et al., 2004; Wu et al., 2010). Although *P. thunbergii* is an outcrossing species, it is known to have the potential to develop a self-fertilizing population by artificial self-pollination (Katsuta, 1964). Lacking a reference sequence for applying a genome-wide GBS approach, we considered that using a self-fertilized S_1_ population would simplify detection of genetic segregation and the locus related to PWD resistance in *P. thunbergii*. Utilizing this approach also broadens the range of genetic approaches for targeted loci detection, and facilitates the further accumulation of genetic knowledge on loci associated with PWD resistance.

In the present study, the GBS approach was used for SNP identification and genotyping of the S_1_ mapping population of the most highly resistant variety of *P. thunbergii*. The identified SNPs were used to construct high-density genetic maps, and SNP genotyping data and phenotyping data for the S_1_ populations were used to identify the locus related to PWD resistance in *P. thunbergii*.

## Materials and Methods

### Plant Material

Sixteen resistant varieties of *P. thunbergii* were selected for the first breeding program and were ranked with regard to resistance (levels 1–5) based on the survival rate of openly pollinated progeny following inoculation with PWN with higher survival rates indicating greater resistance (Toda, 2004). Using self-pollination of the most highly resistant variety, “Namikata 73,” an S_1_ population of 135 progenies was seeded in March 2012 and planted in pots with a diameter of 15 cm and a height of 30 cm in a greenhouse at the Forest Products Research Institute, Forest Tree Breeding Center (FFPRI-FTBC) in Ibaraki, Japan in April 2014. Total genomic DNA of the S_1_ population was extracted from needles using the DNeasy Plant Mini Kit (Qiagen, Hilden, Germany) and subjected to polymorphism analyses, as described below.

### Artificial Inoculation and Phenotyping

The Ka-4 isolate of PWN, which has been used in PWD resistance breeding projects since 2003, was inoculated on the main stems at 5 cm above the ground on 15 June 2014. The bark was shaved with a knife to expose cambium cells, and 5,000 nematodes suspended in 50 μl of sterile water were introduced to the shaved area. Counts of progenies showing resistance (survival) and susceptibility (mortality) were evaluated at 70 days post-inoculation (dpi) with PWN. The count data were subjected to the chi-squared test to assess goodness of fit to the expected Mendelian ratios with a significance threshold of *p* = 0.05.

### Genotyping-by-Sequencing

The GBS library was prepared following a method adapted from Elshire et al. (2011) using 100 ng of genomic DNA per individual and the *ApeK*I restriction enzyme. Individual libraries were barcoded, amplified, and normalized before pooling and sequencing in order to reduce the range in per sample coverage (Nicotra et al., 2016). The GBS libraries of 136 multiplexed samples including the parent sample were sequenced on an Illumina HiSeq 2,500 (Illumina, San Diego, CA, United States) using 100 bp paired-end reads and two lanes by Hokkaido System Science Co., Ltd. (Sapporo, Hokkaido, Japan) and on an Illumina HiSeq 4000 (Illumina) using 100 bp paired-end reads and four lanes by Macrogen (Seoul, South Korea).

A schematic of the progeny sample processing is shown in Supplementary Figure 1. First, four progenies were removed from analysis due to contamination discovered in SSR genotyping, and the sequence data of the parent and 131 progeny GBS raw sequence reads were subjected to a quality check using FastQC (v0.11.2). For SNP calling, samples with target reads that accounted for less than 10% of the mean sequenced reads in the sequencing lane were considered as failed sequencing reactions and removed from analysis (Elshire et al., 2011). In our testing, only two samples were excluded from further consideration for this reason. The final 130 samples (129 S_1_ and one parent) were analyzed using Universal Network Enabled Analysis Kit (UNEAK) pipeline in the TASSEL 3.0 analysis package (Bradbury et al., 2007; Lu et al., 2013). This pipeline permits SNP calling based solely on GBS tag sequence data without requiring a reference genome sequence. Parameters in the UNEAK pipeline to select SNPs were set at a minimum read depth of 5, minimum/maximum minor allele frequencies (MAF, 0.01 and 0.5, respectively), and minimum/maximum call rates (0 and 1, respectively). The MAF and call rate were set at a low values for global analysis because these parameters were filtered within populations at later steps. As filtering was applied before genetic analysis, SNPs that were not heterozygous in the parent genotype and those with a proportion of missing data points greater than 20% were removed from subsequent data analysis. Further, samples showing missing data for more than 20% of all genotypes were removed by manual filtering on Excel.

Raw GBS data, excluding the contaminated progenies, have been submitted to the DDBJ Sequence Read Archive under accession numbers DRA012628, DRA012645, and DRA012646.

A total of 87 genomic DNA-derived SSR markers (Lian et al., 2000; Watanabe et al., 2006; Iwaizumi et al., 2013, 2018; Hirao et al., 2019) were used for segregation analysis in the present study. Multiplex PCR with three or four SSR primer pairs was performed using a Multiplex PCR Kit (Qiagen, Hilden, Germany), with 2× QIAGEN multiplex PCR master mix, 0.25 μm each primer pair, and 40 ng of genomic DNA in a total volume of 10 μl. Amplification was performed on a Veriti thermal cycler (Thermo Fisher Scientific, Waltham, MA, United States) using an initial denaturation step at 95°C for 15 min, followed by 30 cycles of denaturation at 94°C for 30 s, annealing at 57°C for 1.5 min, and extension at 72°C for 1 min, with a final extension at 60°C for 30 min. PCR products (1 μl) were mixed with 0.2 μl GeneScan 500 LIZ size standard (Thermo Fisher Scientific) and 9.8 μl of Hi-Di formamide (Thermo Fisher Scientific) prior to electrophoresis. The length of the amplified fragments was analyzed on an ABI 3130xl sequencer (Thermo Fisher Scientific) and alleles were scored with GeneMapper v5.0 software (Thermo Fisher Scientific). Four samples showing contamination were eliminated from further consideration.

For EST-derived SNP genotyping, we used an array of 768 SNPs on Illumina’s GoldenGate platform (Hirao et al., 2019). Genotyping of the SNP markers was carried out using the custom oligonucleotide pooled assay (OPA; Illumina Inc., San Diego, CA) containing the allele-specific and locus-specific oligos for use in the Illumina GoldenGate assay and Illumina’s BeadArray Express Reader according to standard manufacturer protocol. Automatic allele calling for each locus was inferred with GenomeStudio Software (Illumina).

### Linkage Map Construction

GBS-based genotype data were integrated with the genotyping of SSR markers and EST-derived SNP markers and were applied to genetic analysis. Data could not be obtained for three samples in the GoldenGate assay, and finally, 116 samples were applied to genetic analysis. Linkage analysis was conducted using JoinMap 4.1 (Van Ooijen, 2006) with the genotype data coded as an F_2_ intercross population type. Genotypic data completeness was filtered as 99% on 116 samples, and aberrant segregated loci (*p* < 0.05) were removed by the chi-squared test. Grouping was performed using independent logarithm of odds (LOD) scores from 2 to 20 with a step size of 1, after which the minimum LOD score of 10 was used to determine the autonomous LGs. The groups were converted to maps at LOD using a regression algorithm with the following settings: linkages with recombination frequency (<0.4), LOD (>0.01) threshold for removal of loci with respect to jumps in goodness-of-fit (5.0) and performing a ripple after adding 1 locus. Distance was calculated using Kosambi’s mapping function. A map was drawn using Mapchart 2.32 (Voorrips, 2002) based on the genetic location determined by JoinMap 4.1.

The numbering of the linkage group and the order of the DNA markers in the linkage map constructed in the present study were determined by comparison with a previously constructed linkage map of *P. thunbergii* (Hirao et al., 2019). Based on information of common markers mapped on the two linkage maps, the number and orientation of the linkage group or the relative order and location of the mapped genes in *P. thunbergii* were compared to the consensus linkage map in previous study.

### QTL Analysis

QTL analysis was performed using MapQTL 6.0 software (Van Ooijen, 2009). To perform the binary trait method in the present QTL analysis, resistant and susceptible phenotypes were assigned values of 0 and 1, respectively, and two different methods were used. First, interval mapping (IM) at 1 cM intervals was carried out to detect putative QTL regions. The genome-wide and LG-specific LOD thresholds for each QTL were calculated using a permutation test with 1,000 repetitions at *p* < 0.05 (5%). Closely flanking markers were then selected as cofactors and multiple QTL mapping (MQM) also known as composite interval mapping was performed. The confidence interval for each resulting QTL was determined by a decrease in 1 LOD unit from the QTL LOD peak. Second, the non-parametric Kruskal–Wallis (KW) test module was applied to traits to confirm the significance of the marker within the confidence interval. The allelic effect for the QTL was calculated in MapQTL6 based on the genotypes of loci detected with significance by the KW test using the formula proposed by Knott et al. (1998). Additive and dominance effects, as well as the degree of dominance of the QTLs, were calculated as described in Stuber et al. (1987) and Frérot et al. (2010).

## Results

### Construction of a GBS-SNP-Based Genetic Linkage Map

Barcoded GBS libraries were constructed for 135 *P. thunbergii* progenies and their parent using the enzyme *ApeK*I and pooling for sequencing. Raw data of Illumina sequences of all 136 libraries totaled 1.8 × 10^9^ reads and 177.5 GB, and after removing four samples that were contaminated and two samples with low quality, the raw sequences of 129 progenies and the parent were processed using *de novo* SNP calling and the UNEAK algorithm implemented in TASSEL 3.0. A total of 535,452 SNPs were obtained with the default setting but with the minimum tag count set to five. After application of a series of filters, which removed another 10 progenies, a total of 27,827 high-quality SNPs were obtained from 119 progenies.

Of the 87 genomic SSR markers, genotyping was performed on 131 individuals excluding four contaminated samples. Fifty-four (62.07%) markers showed clear amplification, were heterozygous in the parent genotype and had the possibility of showing expected genetic segregation. Those markers were integrated with EST-derived SNP markers, and GBS-based genotype data for further genetic filtering.

For EST-derived SNP markers using Illumina’s GoldenGate platform, genotyping was performed on 129 individuals, excluding four contaminated samples and two samples with insufficient data in GBS. Firstly, three samples with missing data for more than 20% of all genotypes were removed from subsequent data analysis. Of the 768 loci, 599 SNPs (77.99%) that were not heterozygous in the parent genotype and those with more than 20% missing data points were removed from subsequent data analysis. The genotype data of 169 markers (22.01%) that passed this filtering were integrated into the genomic SSR genotype data and GBS-based genotype data, and further filtering for the expected Mendelian segregation ratio was performed to construct a linkage map.

GBS-based genotype data were integrated with genotyping of SSR markers and EST-derived SNP markers, and this data set was used for genetic analysis. No data could be obtained for three samples with the GoldenGate assay (for genotyping EST-derived SNPs), and we finally used markers from 116 samples for construction of a linkage map. The number of samples in the analysis leading to the construction of the linkage map is shown in Supplementary Figure 1. For construction of the genetic map, distorted markers that did not show the expected 1:2:1 segregation pattern (at a threshold of *p* < 0.05), and markers with data set completeness of less than 99% were removed from those obtained from the 116 samples. A total of 2,397 markers (34 SSR markers, 107 GG markers, and 2,256 GBS-SNP markers) from the remaining 116 progenies were used to construct the genetic map.

**Figure 1:**
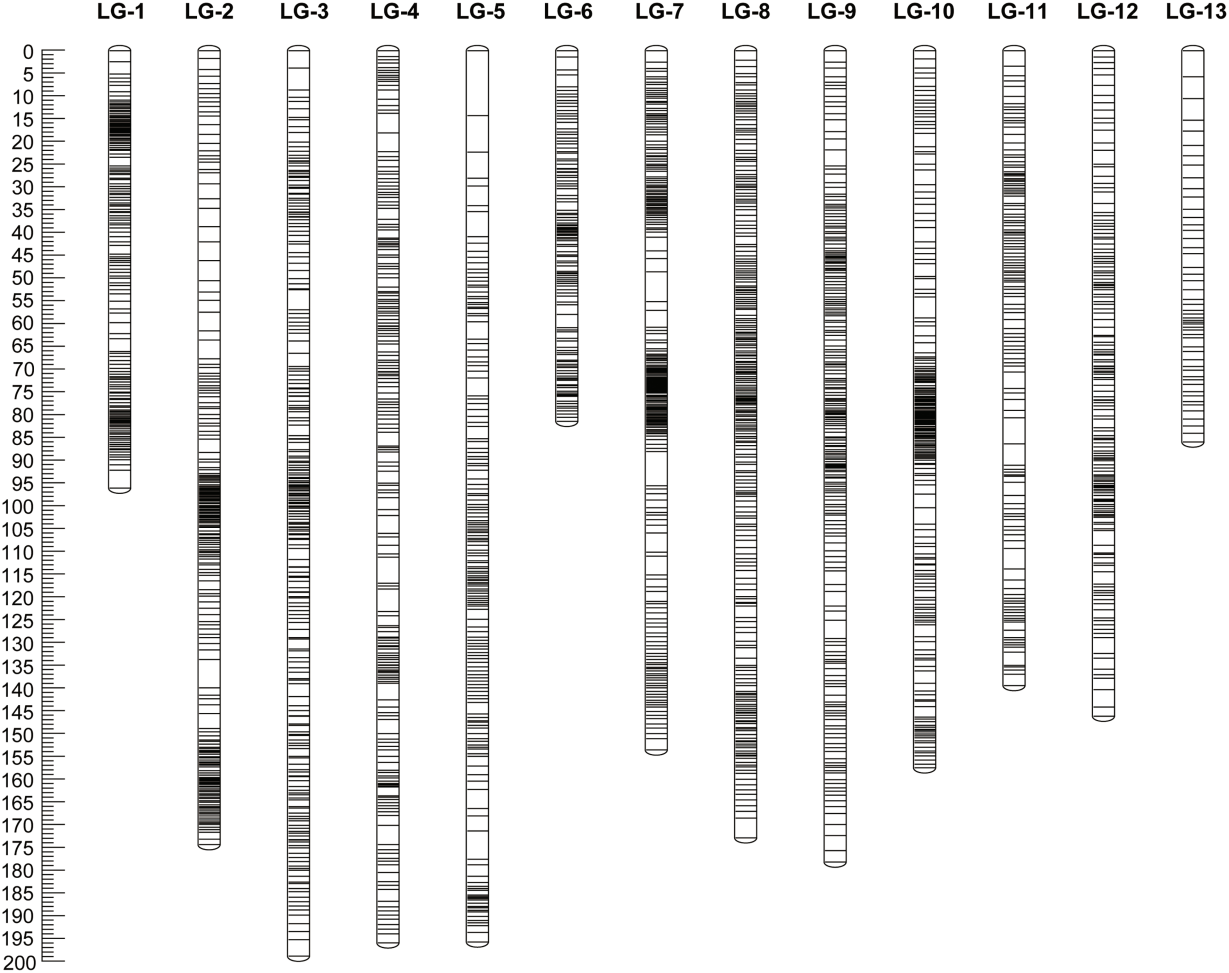
Genetic map constructed in the S_1_ mapping population derived from self-pollination of “Namikata 73.” The map includes 2,365 markers (29 SSR, 93 EST-SNP, and 2,243 GBS-SNP) across 13 LGs for a total length of 1,968.4 cM.

A total of 17 markers (1 genomic SSR marker, 3 EST-derived SNP markers and 13 GBS-SNP markers) of 2,397 markers were not grouped, 15 markers (4 genomic SSR markers, 10 EST-derived SNP markers, and 1 GBS-SNP marker) of 2,380 markers were removed due to having the same similarity loci, and the remaining 2,365 markers (29 SSR markers, 93 GG markers, and 2,243 GBS-SNP markers) were distributed over 13 LGs and had a minimum LOD score of 10.0 (Figure 1; Table 1; Supplementary Tables S1A**–**C). The linkage map spanned a cumulative distance of 1,968.4 centimorgans (cM), with each LG ranging from 81.07 cM (LG-6) and 122 markers to 198.11 cM (LG-3) and 243 markers. The average distance between the markers ranged from 0.59 (LG-1) to 0.88 (LG-5), and the average spacing between two adjacent markers was 0.79 cM across 13 LGs with the largest gap being 14.15 cM (on LG-5).

**Table 1:** Characterization of the genetic linkage map of ‘Namikata 73’.

Linkage group	Number of Markers			Length (cM)	Avg. marker distance (cM)	Max. gap (cM)
	gSSR	EST derived SNPs	GBS-SNPs			
1	1	6	155	95.68	0.59	3.90
2	2	5	201	173.72	0.73	6.20
3	4	8	231	198.11	0.77	4.85
4	5	10	183	195.17	0.82	5.63
5	1	4	163	195.01	0.88	14.15
6	0	7	115	81.07	0.86	2.92
7	2	10	227	153.04	0.82	7.50
8	1	13	242	172.22	0.80	4.31
9	6	8	204	177.49	0.80	4.02
10	0	4	199	156.88	0.80	4.59
11	2	9	122	138.87	0.81	5.76
12	5	7	152	145.56	0.82	3.81
13	0	2	49	85.62	0.84	5.66
Total	29	93	2,243	1968.43	–	–

We compared the constructed linkage map in the present study to the previously constructed a genetic linkage map of *P. thunbergii* (Hirao et al., 2019). The position of 25 genomic SSR markers and 44 EST-derived SNP markers mapped on the *P. thunbergii* linkage map constructed in the present study were used to identify the numbering of the linkage group and the order of the DNA markers. The relative order of mapped markers on the linkage maps for the two types was highly correlated, *R*^2^ = 0.888–0.999 (Supplementary Table S2), although there were not enough markers to compare the relative order of the mapped genes in some linkage groups (LG-10). In addition, since the markers that can be compared to the linkage map of the previous study were not mapped for the 13 linkage groups, it was not possible to verify the original linkage group identification (which linkage group should be integrated with this linkage group) or the order of the DNA markers.

### Evaluation of PWD Resistance in the Population

Although the inoculation test was originally started with 131 S_1_ progenies (see Supplementary Figure 1 for analysis schematic), only 116 progenies were used to construct the genetic linkage map after removing samples due to contamination and poor or missing genotype data for genotyping. Results of the inoculation test and genetic segregation for the 116 progenies used to construct the genetic linkage map are shown in Table 2, Supplementary Table S3, and results for the original 131 progenies are shown in Supplementary Table S4. To test whether the resistance trait is controlled by a single gene, the chi-squared test was applied to phenotypic data of resistant (survival, 1) and susceptible (mortality, 0) states with the null hypothesis that the ratio of susceptible to resistant individuals used to make the linkage map should have a 1:1 segregation ratio. On the other hand, the null hypothesis of a 3:1 ratio of susceptible to resistant individuals was rejected. These results indicate that PWD resistance in the S_1_ population is controlled by a major gene.

**Table 2:** Segregation ratio of PWD resistance in the ‘Namikata 73’ S_1_ mapping population (*n* = 116).

Progeny	Binary standard^**a**^		Total	Expected ratio (1:1)^*^		Expected ratio (3:1)^*^	
	Resistant (Survival; 1)	Susceptible (Mortality; 0)		*X* ^2^	*p*-value	*X* ^2^	*p*-value
S_1_ population	57	59	116	0.03	0.85	36.05	1.927e-9

a Resistant and susceptible are evaluated based on mortality.

* Chi-squared and *p*-values (one degree of freedom) are calculated under the assumption of Mendelian 1:1 and 3:1 segregation ratios.

### Identification of a Locus for PWD Resistance in the NK73-S_1_ Mapping Population

**Figure 2:**
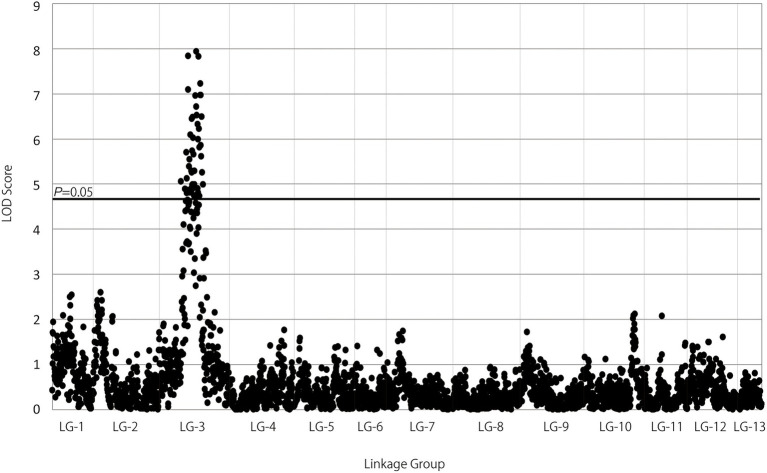
Manhattan plot of interval mapping of LOD scores for the association between DNA marker intervals and phenotype in the NK73S1 mapping population identified significant associations with DNA markers on LG-3. The genome-wide *p* = 0.05 threshold for the data set was determined to be LOD = 4.8 by a permutation test run (*N* = 1,000) on the genetic linkage map.

**Figure 3:**
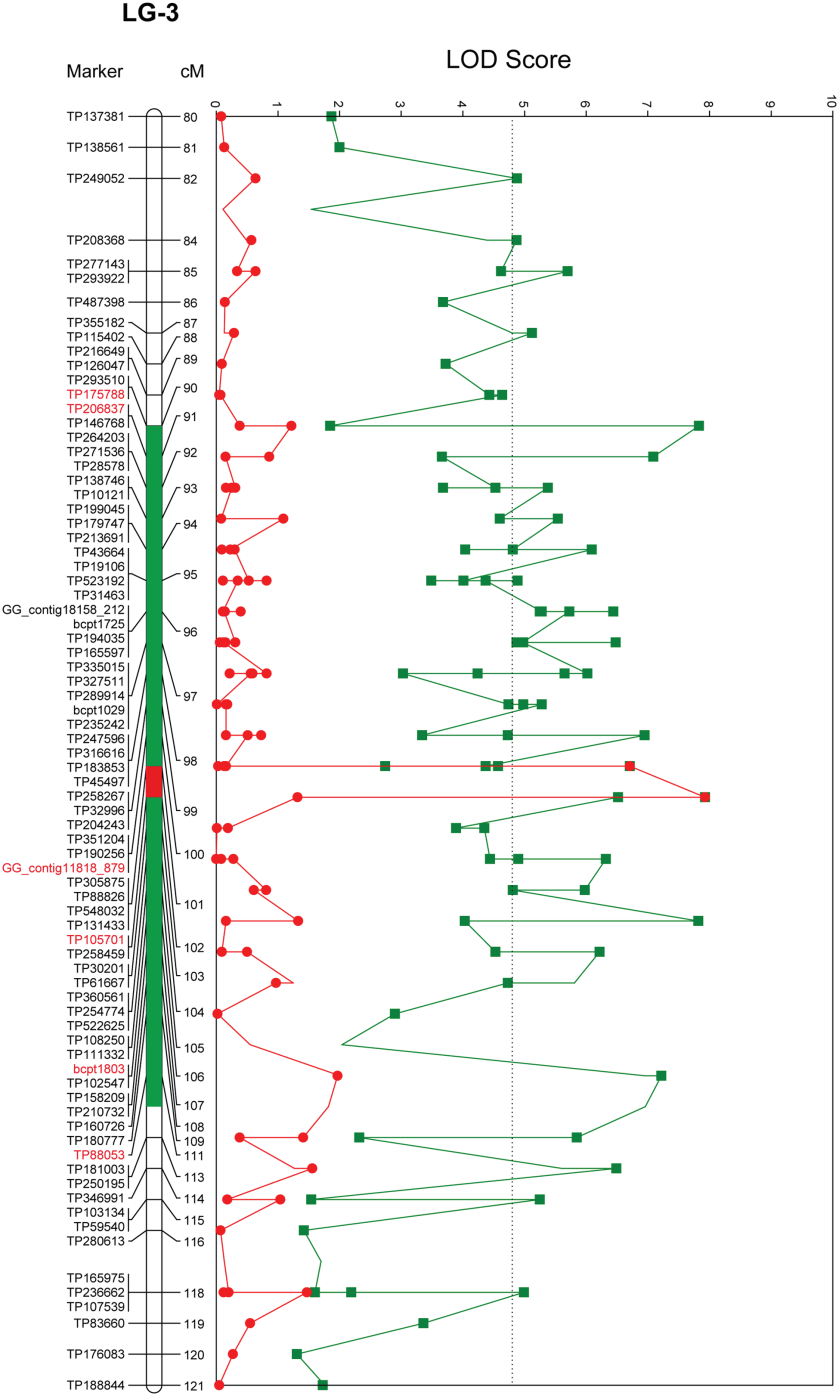
Localization of QTLs for PWD resistance identified in resistant variety “Namikata 73.” Only the area around the QTL was detected are displayed, and the red marker is located in the *PWD1* interval. The line trace in green shows the LOD score determined by interval mapping. The line trace in red shows the LOD score determined by multiple QTL mapping. Green tones and red tone as colored bars indicates a 1-LOD confidence interval of interval mapping and multiple QTL mapping. The genome-wide *p* = 0.05 threshold for the data set was determined to be LOD = 4.8. The genetic linkage maps are in centimorgans (cM).

QTL analysis performed using the constructed genetic linkage map and phenotypic data from a PWN inoculation test revealed a locus for PWD resistance on LG-3 (Figure 2) and a maximum plateau LOD score of 7.9. Permutation tests with 1,000 permutations yielded an LOD score threshold with statistical significance of *α* = 0.05 for PWD resistance of 4.8, which was used as the threshold to detect QTL for PWD resistance with genome-wide significance. MQM mapping confirmed the location of the major QTL that explains up to 27.0% of the total phenotypic variance observed for PWD resistance with a maximum LOD of 7.9 at 101.87 cM on LG-3 (Figure 3; Table 3). Based on all scores, MQM mapping identified marker TP105701 as a cofactor for this region. The QTL regions defined by a 1-LOD confidence interval around a significant QTL LOD peak contained three GBS-SNP markers, one EST-derived SNP marker, and one genomic SSR marker, respectively (Figure 3; Table 3).

**Table 3:** Quantitative trait loci (QTLs) detected for PWD resistance in ‘Namikata 73’ S1 mapping population on the linkage group 3.

LG^a^	Locus	Marker type	Position (cM)	allelej	allele_2	LOD	PVE (%)^b^	K^c^	Signif. KW^d^	Df^e^	Genotype score^f^			Additive	Dominance	Allelic effects^g^
											Mean-hom-allelei	Mean-hetero	Mean-hom-allele2			
3	TP175788	GBS-SNP	90.06	T	A	7.83	26.7	30.729	^*******^	2	3.800	1.931	2.393	−0.704	−1.165	OD (1.656)
3	TP206837	GBS-SNP	90.72	T	C	7.09	24.5	28.209	^*******^	2	3.719	1.981	2.219	−0.750	−0.988	OD (1.317)
3	GG_contig11818_879	EST-derived SNP	100.10	C	T	6.95	24.1	27.733	^*******^	2	3.793	2.034	2.241	0.776	−0.983	OD (1.267)
3	TP105701	GBS-SNP	101.87	C	T	7.93	27.0	31.069	^*******^	2	3.727	1.889	2.345	0.691	−1.147	OD (1.660)
3	bcpt1803	genomic SSR	105.67	235	237	7.82	26.7	30.695	^*******^	2	3.893	2.033	2.222	0.835	−1.025	OD (1.227)
3	TP88053	GBS-SNP	111.33	C	T	7.22	24.9	28.660	^*******^	2	3.793	1.984	2.385	0.704	−1.105	OD (1.569)

a Linkage group.

b Parcentage of the phenotypic variation explained by the QTL.

c Kruskal–Wallis analysis (K^*^) test regarded as nonparametric equivalent of one-way analysis of variance (Van Ooijen, 2009).

d Significant markers ^*******^p < 0.0001.

e Degree of freedom.

f Arithmetic mean of the distribution of the quantitative trait associated with homozygous of allele 1, heterozygous, and homozygous of allele 2, respectively.

g Allelic effects: additive effect (a), dominance effect (d), average degree of dominance (k), allelic interaction, average of disease severity, and mean severity reduction. Average degree of dominance (k) was estimated according to the formula k = |d|/|a|, indicating the following allelic interaction possibilities for a marker associated with QTL: absence of dominance (AD) if k = 0, partial dominance (PD) if 0 < k < 1.0, complete dominance (CD) if k = 1.0, and overdominance (OD) if k > 1.0. (Stuber et al., 1987; Frérot et al., 2010).

**Figure 4:**
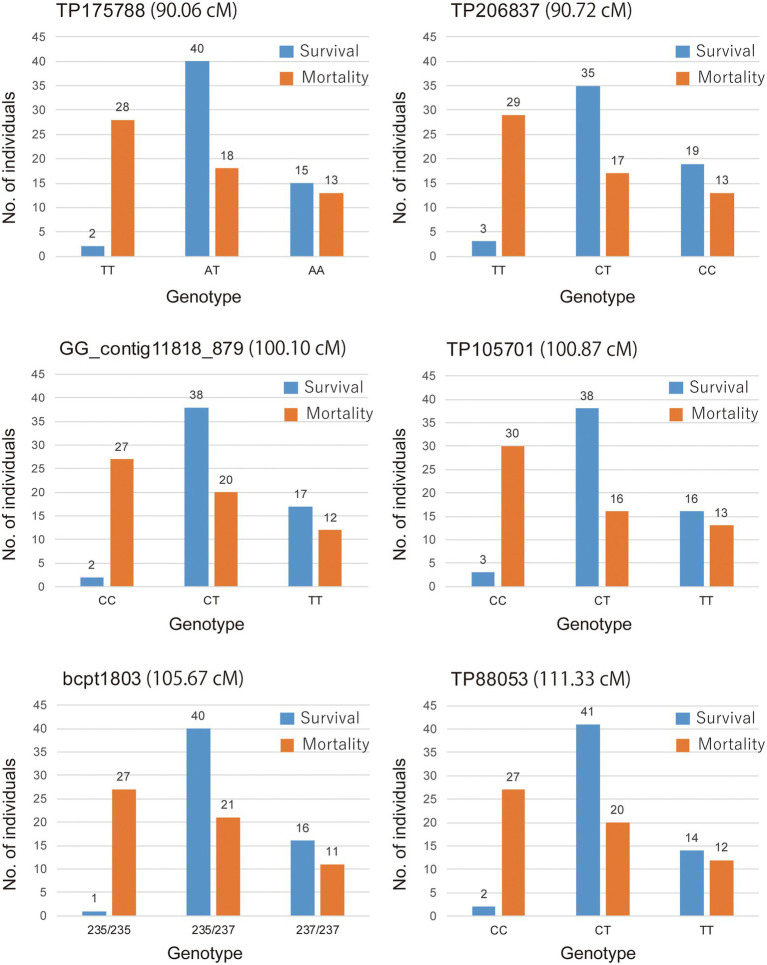
Association between genotype and phenotype of *PWD1* in the NK73 S_1_ mapping population. Number of individuals in the survival and mortality categories for genotypes of marker on the QTL regions defined by a 1-LOD confidence interval.

The marker (TP105701) with a maximum LOD and the other five markers in the QTL regions defined by a 1-LOD confidence interval were converted to the actual detected allele, and those were shown to be significantly associated with the corresponding phenotypic data by the KW test and exhibited an overdominance effect (Table 3). The genotype of the nearest marker (TP105701) corresponded to genotype a (CC), h (CT), and b (TT) respectively. The highest LOD peak marker TP105701 was recorded with genotype 33 “CC”: 54 “CT”: 29 “TT.” Based on the allelic test of marker (TP105701) by the 2 × 2 chi-squared test, the “T” of allele-1 was shown to be significantly associated with PWD resistance while the “C” of allele-2 was associated with PWD susceptibility (*X*^2^ = 15.46, *p* = 0.000084). Similar results were shown for the other five markers (*X*^2^ = 12.533–17.660, *p* = 0.0004–0.000026). The genotype–phenotype relationship was visualized by the real number of progeny for the mortality and survival groups for each locus genotype in a bar graph (Figure 4). These results indicate that the homozygous genotype of allele-1 actually confers susceptibility, but homozygous genotype allele-2 does not confer resistance, and heterozygous genotypes of allele-1 and allele-2 confer resistance, which corroborates the results above that the effect of the allele was detected as an overdominance effect.

## Discussion

The resistant variety “Namikata 73” used in this study is one of the 16 resistant varieties selected from 14,620 candidate trees collected from affected forests of southwest Japan over 7 years from 1978 to 1985 (Fujimoto et al., 1989). In a previous study, the heritability of resistance traits in resistant varieties was evaluated based on the survival rates of open-pollinated progenies with the survival rate of open-pollinated progeny in “Namikata 73” being the highest at 68.9%, compared to 38.5 to 61.1% for other resistant varieties and 12.5% for susceptible pines (Toda and Kurinobu, 2002). Results of previous studies indicate that this variety may carry a highly effective resistance gene/locus, but so far, the locus of this variety has not been clarified by molecular genetic approaches. On the other hand, a very effective defense response against PWN infection by this variety when compared to susceptible trees has been characterized at the transcriptional level (Hirao et al., 2012) as being a moderate defense response mediated by pathogenesis-related protein expression, followed by significant upregulation of cell wall-related genes induced by ROS. These results provide important insights for the resistance or defense response against PWD in other varieties or other *Pinus* species. Thus, identifying the resistance locus of this variety is an important clue for systematically elucidating the resistance mechanism to pine wilt disease.

To date, there have been few reports of linkage mapping in *P. thunbergii*. A genetic linkage map constructed using genomic SSR, EST-derived SSR, or SNP markers for F_1_ mapping with co-dominant markers and showing 12 LGs spanning over 1403.6 cM was recently reported by Hirao et al. (2019). Here, we applied high-throughput GBS technology with type-II restriction endonuclease *ApeK*I (GCWGC; Elshire et al., 2011) to identify SNPs in S_1_ segregated populations for the PWN resistance trait. In addition, genomic SSR markers and EST-derived SNP markers were also applied as anchor markers for comparison purposes with the consensus linkage map of *P. thunbergii* constructed in the previous study (Hirao et al., 2019). Since the draft genome of *P. thunbergii* has not yet been determined, we performed non-reference based GBS with the UNEAK pipeline (Lu et al., 2013), and a total of 2,243 GBS-SNP markers were used to construct 13 LGs spanning over 1,968.43 cM.

When comparing the order of the markers that are commonly mapped on the two maps for the linkage maps constructed in this study and the linkage maps constructed in the previous study by Hirao et al. (2019), the relative order in which markers were mapped on these two linkage maps were highly correlated. Linkage group 10 has not been fully validated as there were not enough markers to compare the relative order of the mapped markers. The length of the linkage map (1,968.43 cM) in the present study was approximately 500–560 cM longer than previously published linkage maps (1469.8 cM in Kondo et al., 2000 and 1403.6 cM in Hirao et al., 2019). GBS used in the present study provided a high mean marker density genetic map of 0.79 markers/cM, which is higher than the 15.6 and 3.26 mean marker density obtained on a previously constructed RAPD (random amplified polymorphic DNA)-based map (Kondo et al., 2000) or microsatellite-and EST-based map (Hirao et al., 2019). We considered that this is due primarily to a larger number of SNP markers being placed on the genetic map.

Although the 13 linkage groups constructed in the present study did not converge to the 12 basic chromosomes of *P. thunbergii*, we can assume that they may constitute one linkage group considering that LG-6 and LG-13 have shorter overall lengths than the other LGs. The EST-derived SNP marker (GG_contig11987_905) on LG-13 could not be associated with the consensus linkage map of *P. thunbergii* constructed in a previous study (Hirao et al., 2019), but we showed correspondence with the locus/gene (0_17469) with high homology for LG-6 in the linkage map of *P. taeda* (Neves et al., 2014; Westbrook et al., 2015; data not shown). LG-6 and LG-13 correspond to the bottom and the upper parts, respectively, of *P. taeda* LG-6. The reason that LG-6 does not converge to one linkage group may be a sufficient number of polymorphisms that could not be detected by the GBS approach with type-II restriction endonuclease *ApeK*I or the presence of a lethal gene.

The genome-wide genotyping and construction of high-density linkage maps is an essential initial step in advancing fundamental genetics research and breeding programs for PWD resistance. Sequencing-based genotyping technology, such as GBS and RADseq, may result in large amounts of missing data, and missing data along with genotyping errors often leads to inaccurate ordering of markers of linkage maps. Although utilizing high-quality SNPs with <20% missing data without imputation has been attempted as an alternative for improving the data quality in a previous study (Liu et al., 2014), we eventually utilized GBS-SNPs with completeness of 99% integrity to construct the linkage map for delineating *PWD1* in the present study. With this approach, we considered that the negative effect caused by missing data could be minimized. Furthermore, we were able to sufficiently narrow down the genetic polymorphisms and particular genome regions that are candidates for PWD resistance based on comparisons with results of previous constructed linkage maps in pine species.

As shown in the present study, PWD resistance in the S_1_ mapping population might be controlled by a major gene, as genetic analysis suggests good fit to a Mendelian ratio of 1:1 with a chi-squared value of 0.03 and probability of 0.85. Regarding the phenotype evaluation, assessment of external wilting symptoms after artificial inoculation is the most commonly used method for evaluating susceptibility and resistance in the host pine, and genetic analysis based on mortality evaluation (survival and mortality) confirmed that PWD resistance is a heritable trait (Toda and Kurinobu, 2001; Kurinobu, 2008; Menéndez-Gutiérrez et al., 2017). Binary data based on mortality were used for the actual QTL analysis in this study, and we succeeded in detecting a major locus that contributes to PWD resistance.

As a result of linkage analysis with the constructed genetic linkage map and phenotypic data in the resistant variety “Namikata 73” S_1_ mapping population, the PWD resistance locus was localized to LG-3 with a map distance of 101.87 cM. In addition to the GBS-SNP marker; TP105701 which has the highest LOD value and the highest contribution to trait, five markers (three GBS-SNP markers, one EST-derived SNP marker, and one genomic SSR marker) were detected as effective markers under the QTL confidence interval on LG-3. These QTLs have been detected over the range of 90.06 to 111.33 cM (interval of approximately 21 cM). The locus associated with PWD resistance detected in the present study was found to be on the same locus (*PWD1*: *PINE WILT DISEASE 1*) as in a previous study (Hirao et al., 2019) by making a comparison with the position of EST-derived SNP markers and genomic SSR markers used for mapping the F_1_ population in a previous study. In that previous study, a major *PWD1* locus for PWD resistance was detected over a confidence interval corresponding to a span of approximately 40 cM on the constructed map of LG-3 and corresponding to the maternal “Tanabe 54” based on maternal segregation. “Tanabe 54” was another one of the resistant varieties selected along with “Namikata 73” for use in the breeding project conducted in the 1970s. While the survival rate of open-pollinated progenies of “Tanabe 54” was 38.5%, it showed the lowest heritability among the other 15 resistant varieties. The results of the present study indicate that the PWD resistance locus is identical to the PWD resistance locus in “Tanabe 54” with low heritability (38.5%) and in “Namikata 73” with high heritability (68.9%), which is an important clue for elucidating genes and their inheritance patterns for PWD resistance in the future.

For GBS-SNP marker TP105701, which has the highest LOD value and the highest contribution to trait, the “C” of the actual allele is clearly shown to be the susceptibility factor in *PWD1* in the present study, the overdominant gene action of resistance conferred by the “T” allele appears in the heterozygous genotype. On the other hand, the resistance effect of the homozygous “TT” genotype in *PWD1* was almost as ineffective as the heterozygous “CT” genotype. The genetic action of this locus was detected as being overdominant according to the degree of dominance in QTL analysis. Overdominance has been recognized as the allelic interaction explanation for hybrid vigor or heterosis; allele “A” and “B” at a locus can interact to cause a superior phenotype compared with both the “AA” and “BB” homozygous states (Birchler et al., 2006; Lippman and Zamir, 2007; Crow, 2010). In the present study, the “C” and “T” alleles at the *PWD1* locus have some interaction, and the heterozygous “CT” genotype exhibited overdominant action for PWD resistance. On the other hand, overdominance is also sometimes actually considered to be pseudo-overdominance. In pseudo-overdominance, repulsion-phase linkage (trans-complementation) of dominant and deleterious genes represent non-additive allelic action and occur when the active parts of a locus are interrupted by inactive parts such that heterozygosity becomes necessary for completion of chain transcription (Jones, 1917; Fasoula and Fasoula, 1983; Birchler et al., 2006; Lippman and Zamir, 2007; Crow, 2010). In *PWD1* of “Namikata 73,” the dominant gene (linked with allele “T”) suppresses the function of the susceptible gene (linked with allele “C”), and the susceptible gene may be linked in the repulsion phase, such that heterozygous S_1_ progenies obtained by self-pollination of “Namikata 73” may have the same high resistance as “Namikata 73” itself. Detailed examination of the genetic hypothesis (e.g., overdominance or pseudo-overdominance and epistatic action) needs to be conducted multi-generationally in order to separate out repulsion-phase linkage.

The other five markers in the QTL confidence interval (3 GBS-SNPs, 1 EST-derived marker, and 1 genomic SSR marker) showed similar trends and effects as TP105701. In addition, many markers have been detected in KW analysis, a non-parametric test. There are 65 markers associated with resistance traits at the level of *p* < 0.0001, including the above 6 markers, including 60 GBS-SNPs, 2 EST-derived SNPs, and 3 genomic SSR markers (Supplementary Table S5). Those GBS markers will be important information in identifying resistance candidate regions in more detail when the whole genome of *P. thunbergii* will have been determined in the future (Supplementary Table S1 and Supplementary File 1). Regarding EST-derived markers, contig11818 has been targeted polymorphisms on genes homologous to phenylalanine ammonia-lyase, and contig18158 has been targeted polymorphisms on genes homologous to hypothetical protein ARALYDRAFT_891246. (See Supplementary Material of Hirao et al. (2019) for the information on the Illumina GoldenGate assay). It is unclear at this time whether these genes are involved in PWD resistance, but markers on highly conserved genes provide important information for comparison with genomic information of other pine species and other tree species. Furthermore, SSR marker shows co-dominant inheritance, robust and high reproducibility, high polymorphism, transferability between species, and low requirements of expertise and instrumentation, which are all characters that make it attractive as a marker. In addition, SSR markers are relatively low cost for genotyping and can be used by small labs. Although population-wide scoring of the SSR markers detected in the present study is a time-consuming, labor-intensive, and costly process, the information gained will support fundamental genetics research and breeding for PWD resistance (See Supplementary Material of Hirao et al. (2019) for information on genomic SSR markers).

As one of the genetic approaches in outbreeding plant species, such as trees, the pseudo-testcross strategy (Grattapaglia and Sederoff, 1994) with pseudo-testcross linkage analyses for each of two parents in an F_1_ full-sib family is used. In a previous study, inheritance patterns of PWD resistance traits were investigated by a two-way pseudo-testcross strategy using an F_1_ mapping population derived from resistant varieties cross, “Tanabe 54” × “Tosashimizu 64” (Hirao et al., 2019). Although linkage analysis for PWD resistance using an S_1_ mapping population was first attempted in *P. thunbergii* in the present study, the PWD resistance trait was clearly segregated, and the GBS-derived SNP markers were closely mapped to *PWD1* on LG-3. These results suggest a genetic approach using a self-pollinated family as one of the simplest and most effective approaches for detecting a major locus related to PWD resistance.

The result that the resistance locus was localized to LG-3 in both “Namikata 73” and “Tanabe 54,” the varieties with the highest and lowest heritability, respectively, for progeny for resistance is an unexpected new finding. In order to clarify the genetic mechanism at this locus, it is necessary to obtain genomic information of *P. thunbergii* and to clarify the genomic structure and genetic variation in this QTL region and to furthermore verify its heredity over multiple generations. Furthermore, in addition to the genome-wide genotyping system used in this study, the use of a large single cross to build genetic maps is also an important factor for constructing a high-density and high accuracy linkage map, which has been shown to be effective in the study of other tree species (Bartholomé et al., 2015; Pavy et al., 2017). By developing these verifications, it is possible to develop a more accurate marker system related to resistance traits for realizing resistance breeding by GAS and MAS, and we can thus advance PWN-resistant breeding in *P. thunbergii*.

## Conclusion

In this study, we constructed a high-density linkage map for *P. thunbergii* using GBS-SNP markers, along with anchor markers, such as genomic SSR markers and co-dominant SNP markers derived from ESTs. The use of a high-density sequence-based genetic map facilitated anchoring the QTLs to a physical map and provided a means for making an alignment with previously reported QTLs. Further, the locus for PWD resistance was identified on LG-3 by QTL analysis of the S_1_ mapping population using the constructed genetic linkage map and phenotypic data from a PWN inoculation test. The QTL on LG-3 was co-located with *PWD1*, which was detected in a previous study. The high-resolution genetic map of the S_1_ mapping population also enabled identification of QTLs in a narrower physical interval than for QTLs previously identified with SSR markers. Further, the susceptibility gene and the gene that suppresses the susceptibility gene were suggested to be “pseudo-overdominance” linked in the repulsion phase, although the genetic pattern at this locus was determined to be overdominance. This study thus provides an important clue for elucidating genes and their inheritance patterns for PWD resistance in the future.

## Data Availability Statement

The datasets presented in this study can be found in online repositories. The names of the repository/repositories and accession number(s) can be found in the article/Supplementary Material.

## Author Contributions

TH conducted sampling and inoculation tests, as well as evaluation of phenotypes, prepared DNA, genotyped each DNA marker, constructed linkage maps, performed QTL analysis, and drafted the manuscript. KM mapped population development and seed materials. KS generated GBS genotyping data and constructed linkage maps. All authors assisted with manuscript preparation and read and approved the final manuscript.

## Funding

This research was supported financially by the Ministry of Agriculture, Forestry and Fisheries of Japan as part of the “Project to advance the development of technology for varieties of Japanese black pine and red pine resistant to the pine wood nematode.” This work was also supported by JSPS KAKENHI Grant Number 16K07792.

## Conflict of Interest

The authors declare that the research was conducted in the absence of any commercial or financial relationships that could be construed as a potential conflict of interest.

## Publisher’s Note

All claims expressed in this article are solely those of the authors and do not necessarily represent those of their affiliated organizations, or those of the publisher, the editors and the reviewers. Any product that may be evaluated in this article, or claim that may be made by its manufacturer, is not guaranteed or endorsed by the publisher.

## Supplementary Material

Supplementary Figure 1 Outline of the sample used for the construction of the linkage map and 731 QTL analysis in this study.
