# Supplementary figures and images for: Quantitative Trait Loci Analysis Based on High-Density Mapping of Single-Nucleotide Polymorphisms by Genotyping-by-Sequencing Against Pine Wilt Disease in Japanese Black Pine (Pinus thunbergii)
Source: Front Plant Sci. 2022 Apr 5;13:850660. doi: 10.3389/fpls.2022.850660 (PMC9022113; doi:10.3389/fpls.2022.850660)

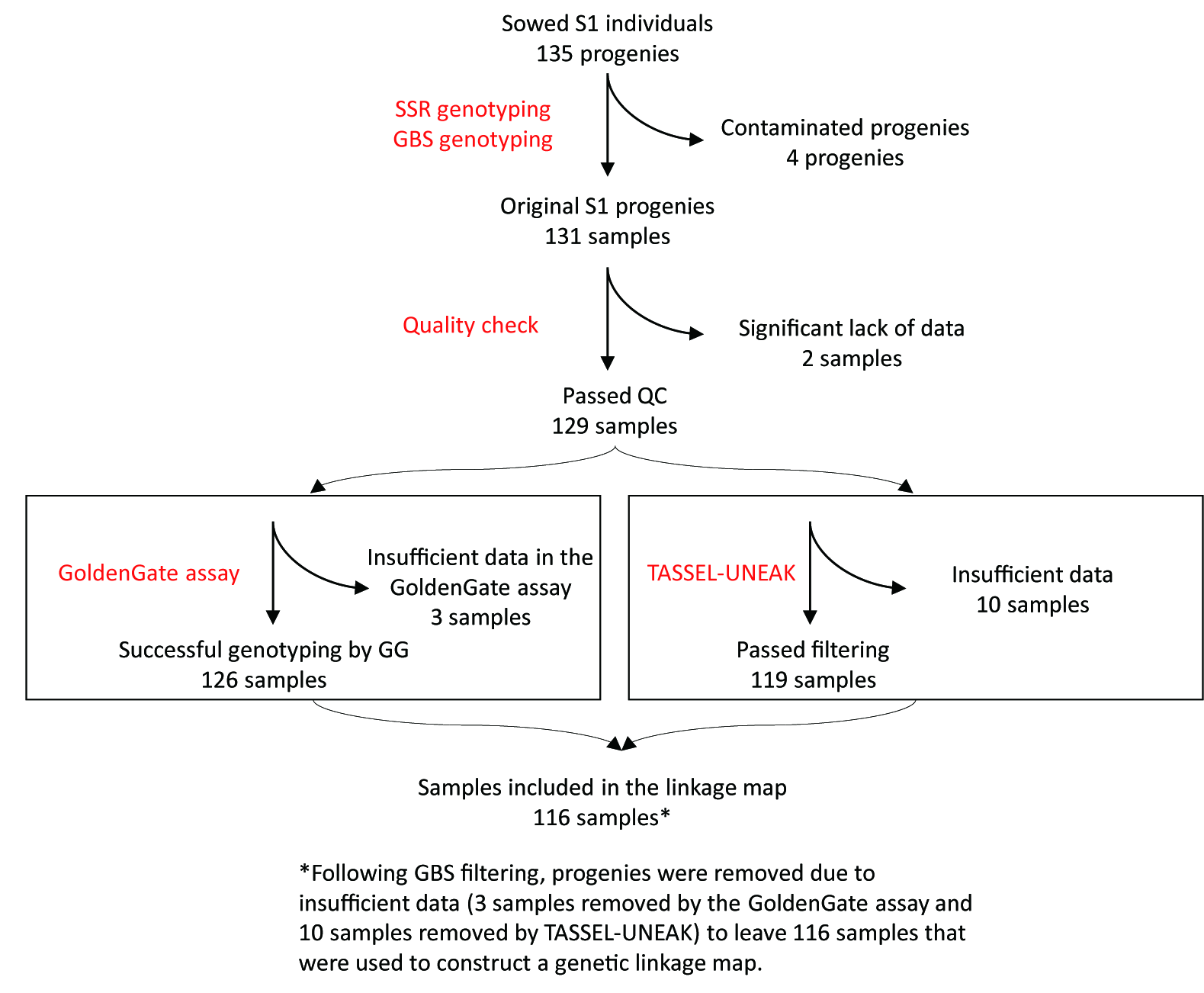

Supplement: Supplementary Figure 1 — Outline of the sample used for the construction of the linkage map and 731 QTL analysis in this study. [file Image_1.TIF]
